# Carotid artery stenting in JAK2 V617F-positive essential thrombocythemia with symptomatic internal carotid artery stenosis: a case report

**DOI:** 10.3389/fcvm.2025.1658456

**Published:** 2025-08-25

**Authors:** Minghui Du, Yinbao Hu, Zhigang Liang

**Affiliations:** ^1^ Department of Neurology, Yuhuangding Hospital Affiliated to Qingdao University, Yantai, China; ^2^ The Second Clinical Medical College of Binzhou Medical University, Yantai, China; ^3^ Shandong Provincial Key Laboratory of Neuroimmune Interaction and Regulation, Yantai, China; ^4^ National Clinical Medical Research Center for Neurological Diseases Regional Subcenter, Yantai, China

**Keywords:** essential thrombocythemia, JAK2 v617F mutation, symptomatic internal carotid artery stenosis, carotid artery stenting, dual antiplatelet therapy

## Abstract

Essential thrombocythemia (ET) is a myeloproliferative neoplasm (MPN) characterized by abnormal megakaryocyte proliferation and a markedly elevated platelet count, which predisposes patients to thrombotic or hemorrhagic events. Approximately 50%–60% of ET patients harbor a JAK2 V617F mutation. This mutation drives constitutive JAK kinase activation, promoting megakaryocyte proliferation and platelet production, while potentially activating inflammatory pathways and damaging vascular endothelium. We report a case of a JAK2 V617F-positive ET patient (sporadic presentation) who successfully underwent carotid artery stenting (CAS) for symptomatic internal carotid artery (ICA) stenosis. A 66-year-old male with known JAK2 V617F-positive ET presented with transient slurred speech and right-sided facial droop with mouth deviation. Magnetic resonance imaging/magnetic resonance angiography (MRI/MRA) revealed an acute cerebral infarction in the right basal ganglia and corona radiata, along with right ICA stenosis. Aggressive perioperative platelet and inflammation control, employing hydroxyurea, aspirin, and ticagrelor, was instrumental in mitigating the heightened thrombosis risk associated with the JAK2 V617F mutation. This case underscores that ET patients with the JAK2 V617F mutation face a substantial risk of thrombotic recurrence. It highlights the critical importance of rigorous preoperative platelet control, personalized antiplatelet therapy guided by pharmacogenomic principles, and multidisciplinary management in high-risk ET patients undergoing CAS.

## Introduction

ET is a myeloproliferative disorder characterized by persistently elevated platelet counts, susceptibility to thrombotic and hemorrhagic complications, and an increased predisposition to arterial and venous thrombosis, which can manifest as arterial stenosis (1). The Janus kinase 2 (JAK2) V617F mutation is associated with inflammation and thrombosis and has been shown to independently increase the risk of ischemic vascular events (2). Artery stenosis represents a significant cause of ischemic stroke in ET patients. However, managing endovascular treatment poses challenges due to elevated platelet counts and bleeding risk. We present a case of successful multidisciplinary management in a high-risk ET patient with the JAK2 V617F mutation who underwent CAS for symptomatic extracranial ICA stenosis. This report emphasizes the importance of perioperative platelet control and an optimized antiplatelet strategy.

## Case presentation

A 66-year-old man presented to our outpatient department with a 4-day history of transient slurred speech, right-sided facial drooling and mouth deviation. His medical history included type 2 diabetes, essential thrombocythemia, an ischemic stroke 6 years prior with no residual deficits and lung tumor surgery 2 years ago. Head magnetic resonance imaging (MRI) revealed acute cerebral infarction in the right basal ganglia and corona radiata. Neck magnetic resonance angiography (MRA) demonstrated right ICA stenosis and carotid artery ultrasound confirmed bilateral carotid atherosclerosis with plaque formation. Following admission, treatment was initiated with hydroxyurea (HU; 500 mg/day), aspirin (ASA; 100 mg/day), ticagrelor (180 mg/day), atorvastatin (20 mg/day) and hypoglycemic agents. Ticagrelor was selected over clopidogrel based on CYP2C19 gene testing indicating slow metabolism. Given that the bleeding risk of ticagrelor is higher than that of clopidogrel, 90 mg bid was chosen over the standard 180 mg loading dose to balance antithrombotic and bleeding risks.

**Figure 1:**
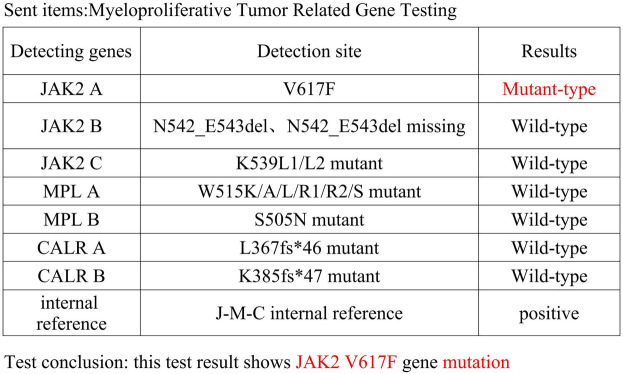
Myeloproliferative neoplasms related gene testing.

Physical examination revealed no significant abnormalities and the patient was hospitalized. Admission routine blood tests showed a platelet (PLT) count of 713 × 10^9^/L (reference range: 125–350 × 10^9^/L) and a white blood cell (WBC) count of 12.67 × 10^9^/L (reference range: 3.5–9.5 × 10^9^/L). MRI demonstrated malacic foci in the left corona radiata and basal ganglia region, as well as the right cerebellar hemisphere, along with multiple white matter hyperintensities suggestive of chronic microvascular ischemic changes. Bone marrow puncture revealed megakaryocytic hyperplasia and thrombocytosis. Cytogenetic studies, including BCR-ABL fusion gene testing and chromosome karyotype analysis, were normal. Reticulin staining (Gomori) showed grade MF-1 fibrosis. A myeloproliferative neoplasm-associated gene panel detected a JAK2 V617F mutation (Figure 1). Bone marrow biopsy confirmed these findings, showing an elevated granulocyte-to-erythroid ratio (G:E ratio) and scattered megakaryocytes with increased platelet production. The granulocyte compartment constituted 69% of the marrow cellularity. The overall bone marrow findings supported the diagnosis of Essential Thrombocythemia (ET). Following consultation with Hematology, the hydroxyurea dose was increased to 1,000 mg/day. High-dose hydroxyurea (1,000 mg/day) may exacerbate myelosuppression, and the risk of platelet plunge needs to be guarded against in combination with DAPT. To avoid excessive inhibition, platelet counts were monitored every two days until reaching the target level of less than 600 × 10^9^/L. We used both the HAS-BLED score (2 points) and ISTH-BAT score (0 points, no history of abnormal bleeding) to further quantify bleeding risk.

**Figure 2:**
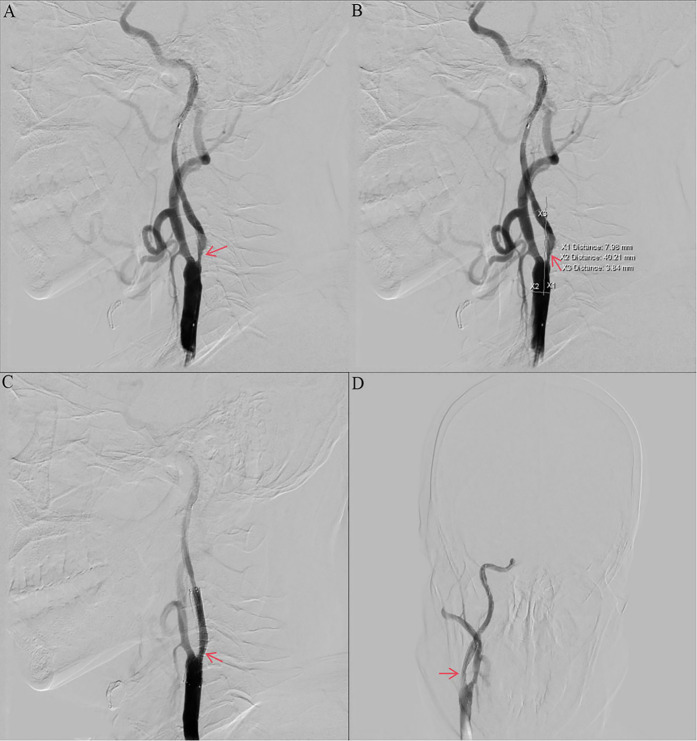
Preoperative and postoperative digital subtraction angiography. **(a, b)** Angiography of the right ICA before CAS showing stenosis with ulcers (NASCET 90% stenosis). **(c, d)** Angiography of the right ICA after CAS showing no residual stenosis and no thrombosis. ICA, internal carotid artery; CAS, carotid artery stenting; NASCET, North American Symptomatic Carotid Endarterectomy Trial; DSA, digital subtraction angiography.

Other auxiliary investigations—including blood glucose, blood lipids, liver and renal function tests, coagulation profile, urinalysis, and thyroid antibody analysis—were unremarkable. Cardiac ultrasound and electrocardiogram findings were also normal. Vascular Surgery consultation recommended carotid endarterectomy (CEA) under general anesthesia. However, the patient and his family declined the procedure due to concerns regarding the risks associated with general anesthesia and the required neck incision. The patient decided to undergo internal carotid artery stenting under local anesthesia. Preoperative routine blood count (obtained two days prior to surgery) revealed a platelet count of 574 × 10^9^/L. Intraoperative angiography demonstrated approximately 90% stenosis within the C1 segment of the right internal carotid artery (Figures 2A,B). The vessel diameter distal to the stenosis measured approximately 2.5 mm, while the common carotid artery diameter was approximately 7.9 mm. The stenosis was pre-dilated using a 4 mm × 30 mm LitePAC balloon inflated to 8 atmospheres(Atm). Subsequently, a 9 mm × 40 mm PROTEGE stent was successfully deployed across the stenotic segment. Post-procedural imaging showed improved antegrade flow with residual stenosis estimated at approximately 10%. Routine blood count on postoperative day 1 documented a platelet count of 415 × 10^9^/L.

The patient received dual antiplatelet therapy (aspirin and ticagrelor) and statin therapy. A stent was successfully implanted in his right internal carotid artery (Figures 2C,D). The CRUSADE scoring system was used to dynamically assess the risk of bleeding during hospitalization (in this case, the score was 24, which is low-risk), and hemoglobin alert values were set (immediate intervention for a drop of >2 g/dl). No recurrent cerebral infarction occurred. He was discharged on hospital day 19 with a modified Rankin Scale (mRS) score of 1. His discharge regimen included continued aspirin, ticagrelor, statin, and hydroxyurea. Discharge laboratory results showed a platelet count of 317 × 10^9^/L (within normal range). Based on the platelet count trajectory and consultation with Hematology, the hydroxyurea dose was reduced to 500 mg/day. At the 1-month follow-up, his symptoms had significantly improved, with a platelet count of 471 × 10^9^/L. Three-month follow-up included carotid ultrasound and platelet assessment (408 × 10^9^/L). The ultrasound demonstrated a patent right carotid stent with smooth blood flow (velocity of 78 cm/s) and no evidence of restenosis. Following completion of 3 months of dual antiplatelet therapy (DAPT) and in accordance with ISTH Antithrombotic Guidelines for Myeloproliferative Neoplasms, ticagrelor was discontinued and antiplatelet therapy was transitioned to aspirin monotherapy. Throughout follow-up, the patient exhibited no progression to other hematologic complications associated with ET.

## Discussion

A platelet count of 450 × 10^9^/L or higher with thrombocythemia is the primary diagnostic criterion for essential thrombocythemia as defined by the International Consensus Classification of Myeloid Neoplasms and Acute Leukemia as well as the World Health Organization criteria (3, 4). The incidence of primary thrombocythemia in adults in the United States is 1.55 per 100,000 population (5). In a retrospective study of 1,076 patients with essential thrombocythemia, 21% developed new thrombosis after a median follow-up of 10 years, with 15% having arterial thrombosis and 8% having venous thrombosis (6).

Approximately 60% of patients with essential thrombocythemia express the JAK2 V617F mutation (7). JAK2 V617F mutation is associated with significantly iIncreased risk of thrombosis (8–10). Aspirin is mostly used for antithrombotic therapy, especially in patients with the JAK2 V617F mutation (11). Once-daily aspirin therapy is usually recommended for most patients with primary thrombocythemia to reduce the risk of arterial thrombosis and venous thrombosis. However, aspirin therapy may not be needed in very low-risk patients without cardiovascular risk factors or in patients without expression of JAK2, CALR, or MPL variants (12–15). Patients at high risk for ET should also have HU as the first-line cytolytic agent of choice to minimize the risk of thrombosis (starting dose of 500 mg once daily) (16). In patients with ET who remain at risk of thrombosis despite aspirin therapy, the addition of hydroxyurea or other cytostatic agents may reduce the risk of recurrent thrombosis by 50% (17, 18). JAK2 V617F mutation promotes pro-inflammatory cytokine release, exacerbates endothelial damage and leukocyte infiltration, and increases the risk of atherosclerosis, requiring statin lipid-lowering therapy. The paradoxical coexistence of both thrombotic and hemorrhagic tendencies in ET, particularly with the JAK2 V617F mutation, is a critical clinical challenge. This phenomenon is primarily attributed to platelet dysfunction and Acquired von Willebrand Syndrome (AvWS). The combined effect of excessive platelet-mediated consumption and accelerated degradation due to endothelial dysfunction and shear stress results in a qualitative and quantitative deficiency of vWF, particularly the loss of HMW multimers. This manifests as AvWS. While vWF antigen levels may be normal or elevated, the functional activity (vWF:RCo) is disproportionately low, and multimer analysis shows the characteristic loss of the largest multimers.

Our patient was considered to be at high risk for thrombosis because of his age >60 years, history of stroke and diabetes, and the JAK2 V617F mutation, which should be treated with a combination of low-dose ASA and hydroxyurea. Clopidogrel inhibits platelets after activation by CYP2C19 metabolism. However, patients with slow CYP2C19 metabolism have reduced activation, leading to decreased drug efficacy and adverse events such as in-stent thrombosis. Ticagrelor is a direct-acting P2Y12 receptor antagonist that is not dependent on the CYP2C19 enzyme, and the ACC/AHA guidelines recommend the use of ticagrelor in patients who are slow metabolizers of CYP2C19. In this patient with CYP2C19 slow metabolism, the combination of aspirin and ticagrelor was chosen to provide more reliable and potent platelet inhibition compared to clopidogrel, aiming to minimize stent thrombosis risk. The observed outcome (no thrombosis, no major bleeding) supports the feasibility and potential benefit of this approach in such high-risk scenarios. This means that more research is needed to identify better treatment strategies. Dual antiplatelet combined with hydroxyurea did not increase the risk of bleeding, possibly related to normal VWF activity.

The vascular surgery team recommends CEA as the first-line revascularization procedure for patients with symptomatic high-grade carotid artery stenosis (≥70%), based on robust evidence. CEA provides direct surgical access to diseased arterial segments, enabling complete removal of unstable, ulcerated atherosclerotic plaques and offering superior immediate control over thrombotic complications during this critical phase. Additionally, CEA avoids implanting a foreign body (stent) in a hypercoagulable environment and typically requires only short-term single antiplatelet therapy perioperatively. Compared to CEA under general anesthesia, CAS offers significant advantages in patients with prothrombotic conditions such as essential thrombocythemia. Performing CAS under local anesthesia eliminates thrombosis risks associated with airway manipulation during intubation. Its minimally invasive nature substantially reduces tissue trauma, thereby attenuating platelet activation and perioperative bleeding risks. Furthermore, rapid postprocedural mobilization mitigates the likelihood of deep vein thrombosis (DVT) secondary to prolonged immobilization. The patient's refusal of CEA was driven by concerns over general anesthesia risks and surgical trauma, making CAS the optimal alternative under meticulous platelet control. Platelets should be controlled in a safe range by stepwise cytoreductive therapy preoperatively to avoid intraoperative thrombosis and bleeding. For aspirin-refractory cases, the target platelet count should be the level at the time of symptom resolution, not necessarily 400 × 10^9^/L (19). While the optimal target platelet count for stroke prevention in ET remains an area of investigation, this case demonstrates that individualized cytoreductive therapy to achieve a significant reduction towards safer levels (400–600 × 10^9^/L), combined with tailored antithrombotic strategies and appropriate revascularization, can lead to successful outcomes in high-risk patients with symptomatic stenosis.

## Conclusions

Essential thrombocythemia (ET), a myeloproliferative neoplasm frequently driven by JAK2 V617F mutations, results in thrombocytosis and hypercoagulability, elevating the risk of bleeding and thrombotic events including ischemic stroke. Multidisciplinary coordination (neurology, hematology, vascular surgery) is paramount for developing individualized management. This includes cytoreduction (hydroxyurea), tailored antithrombotic therapy (initially aspirin plus ticagrelor, transitioning to aspirin monotherapy per guidelines), and endovascular revascularization (carotid artery stenting, CAS). Guided by CYP2C19 pharmacogenetic testing, ticagrelor was selected over clopidogrel to ensure effective platelet inhibition in this slow metabolizer. Critically, achieving a platelet count <600 × 10^9^/L pre-intervention is essential. AvWS screening and dynamic monitoring are not omitted safety prerequisites when JAK2 V617F-positive ET patients undergo DAPT. With rigorous screening of patients (normal vWF activity and no bleeding history) and close monitoring, an individualized DAPT (low-dose ticagrelor plus aspirin) combined with hydroxyurea strategy optimizes thrombotic protection while maintaining a favorable bleeding risk profile, achieving a balance between thromboprophylaxis and bleeding risk.

## Data Availability

The datasets presented in this article are not readily available because of ethical and privacy restrictions. Requests to access the datasets should be directed to the corresponding author.
